# Proteomic comparison of the cytosolic proteins of three *Bifidobacterium longum *human isolates and *B. longum *NCC2705

**DOI:** 10.1186/1471-2180-10-29

**Published:** 2010-01-29

**Authors:** Julio Aires, Patricia Anglade, Fabienne Baraige, Monique Zagorec, Marie-Christine Champomier-Vergès, Marie-José Butel

**Affiliations:** 1 Université Paris Descartes, EA 4065, Faculté des Sciences Pharmaceutiques et Biologiques, Paris, France; 2 INRA, FLEC, UR309, Domaine de Vilvert, 78350 Jouy en Josas, France

## Abstract

**Background:**

Bifidobacteria are natural inhabitants of the human gastrointestinal tract. In full-term newborns, these bacteria are acquired from the mother during delivery and rapidly become the predominant organisms in the intestinal microbiota. Bifidobacteria contribute to the establishment of healthy intestinal ecology and can confer health benefits to their host. Consequently, there is growing interest in bifidobacteria, and various strains are currently used as probiotic components in functional food products. However, the probiotic effects have been reported to be strain-specific. There is thus a need to better understand the determinants of the observed benefits provided by these probiotics. Our objective was to compare three human *B. longum *isolates with the sequenced model strain *B. longum *NCC2705 at the chromosome and proteome levels.

**Results:**

Pulsed field electrophoresis genotyping revealed genetic heterogeneity with low intraspecies strain relatedness among the four strains tested. Using two-dimensional gel electrophoresis, we analyzed qualitative differences in the cytosolic protein patterns. There were 45 spots that were present in some strains and absent in others. Spots were excised from the gels and subjected to peptide mass fingerprint analysis for identification. The 45 spots represented 37 proteins, most of which were involved in carbohydrate metabolism and cell wall or cell membrane synthesis. Notably, the protein patterns were correlated with differences in cell membrane properties like surface hydrophobicity and cell agglutination.

**Conclusion:**

These results showed that proteomic analysis can be valuable for investigating differences in bifidobacterial species and may provide a better understanding of the diversity of bifidobacteria and their potential use as probiotics.

## Background

Bifidobacteria are anaerobic high G + C Gram-positive bacteria that belong to the *Bifidobacterium *genus, which contains more than 30 species. *Bifidobacterium *is a prevalent bacterial genus in the human colon that represents up to 90% of all bacteria in fecal samples of breast-fed infants and 3 to 5% of adult fecal microbiota [1,2]. In full-term breast-fed infants, the intestinal microbiota is rapidly dominated by bifidobacteria that are acquired from mothers' microbiota during birth. These bacteria contribute to the establishment of healthy intestinal ecology and can confer health benefits to their host. Indeed, impairment of bifidobacterial colonization is a risk factor for allergic diseases [3] and for necrotizing enterocolitis in preterm infants [4]. Consequently, bifidobacteria are the subject of growing interest due to their assumed contribution to the maintenance of gastrointestinal health [5-12]. For these reasons, some bifidobacterial strains are used as health-promoting or probiotic components in functional food products [13].

Although bifidobacteria have been reported to exert a number of positive biological effects, there has been limited research into the molecular mechanisms underlying these effects. This may be due in part to reports that some of the positive biological activities of bifidobacteria are strain-dependent [14] and to the limited number of sequenced genomes. Applying genomics to bifidobacteria is essential for a better understanding of their effects. Indeed, comparative genomic studies of the few sequenced genomes of bifidobacteria has contributed to a better understanding of the stress response [15,16], bacterial phylogeny and ecological adaptation [16,17], and genetic variability [16,18]. Within the *Bifidobacterium *genus, the first completed genome sequence was that of the probiotic strain *B. longum *NCC2705, which became available in 2002 [16] and was revised in 2005 (GenBank database accession no. AE014295). Recently, the assembled genome of *B. longum *DJO10A became available in the NCBI database (NCBI source NZ_AABM00000000), allowing this genetic information to be used for comparisons and functional analyses such as proteomic comparisons.

Unlike genome studies, investigations at the proteomic level provide insights into protein abundance and/or post-transcriptional modifications. Proteomic studies of the *Bifidobacterium *genus have established reference maps [19,20]; comparisons of differentially expressed proteins have shed light on bacterial adaptations to gastrointestinal tract factors such as bile [21,22] and acidic pH [23]. Although two-dimensional electrophoresis (2D-electrophoresis) has been used to analyze bacterial protein polymorphisms and to distinguish between closely related pathogenic organisms [24-26], 2D-electrophoresis has not been used to compare bifidobacteria.

In this study, our objective was to compare three human *B. longum *isolates with the model sequenced strain *B. longum *NCC2705 at the chromosome and proteome levels. Pulse-field gel electrophoresis (PFGE) revealed a high degree of heterogeneity. Moreover, the isolates showed different patterns in terms of their cytoplasmic proteins that may reveal correlations with specific phenotypic differences of the *B. longum *strains. Our results show that this approach is a valuable tool for exploring the natural diversity and the various capabilities of bifidobacteria strains.

## Results and Discussion

In the present study, we chose *B. longum *NCC2705 as the reference strain because (i) *B. longum *is one of three species used as probiotics; (ii) the entire genome sequence is available, allowing protein identification using a public database [16]; (iii) a proteome reference map had been established for this strain [19]. Three *B. longum *human isolates with known biological effects were compared to this reference strain. In an animal model, *B. longum *BS89 has a protective role against necrotizing enterocolitis via a sharp decrease of clostridia [27]. The two other isolates show differences in their abilities to stimulate the intestinal immune system in gnotobiotic mice by inducing either T-helper 2 (*B. longum *BS64) or T-helper 1 cytokines (*B. longum *BS49) [28].

### Genotype comparison using PFGE

**Figure 1:**
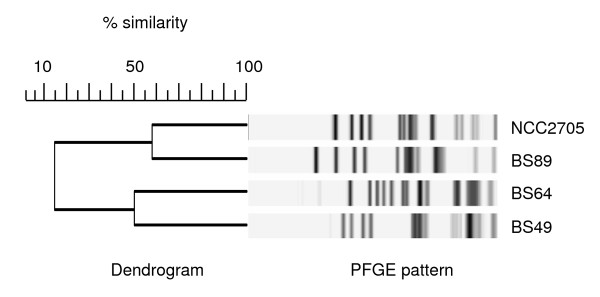
**Comparison of *B. longum *genomic DNA XbaI macrorestriction patterns using pulsed field gel electrophoresis (PFGE) genotyping**.

We first compared the four strains at the genome level using PFGE [29]. XbaI macro-restriction analysis of genomic DNA from *B. longum *strains NCC2705, BS49, BS64 and BS89 generated clear and easy-to-interpret PFGE patterns (Figure 1). The four strains exhibited a high degree of genomic heterogeneity and low intraspecies relatedness: BS89, BS49 and BS64 shared 57.9, 29.3 and 20.9% identity, respectively, with NCC2705 macrorestriction patterns. Such genetic variability is consistent with the comparative genomic analysis of *B. longum *strains NCC2705 and DJO10A, which showed substantial loss of genome regions, probably due to multiple phage insertion sites [18,30]. Considering the various biological effects and genomic heterogeneity of the isolates, one might speculate that this heterogeneity could be related to functional differences that could be identified using proteomic analysis.

### Comparison of cytosolic protein patterns of the *B. longum *strains

We next used 2D-electrophoresis to analyze the cytosolic protein content of these four strains. Spot differences between the three human isolates, BS89, BS49 and BS64, and *B. longum *NCC2075 are summarized in Table S1 (Additional file 1). A total of 45 spots (Additional file 2), representing 37 different proteins, were present in some strains and absent in others. The 38 proteins fell mainly into the following functional categories: (i) metabolism-related proteins, especially proteins related to cell wall/membrane/envelope biogenesis; (ii) proteins involved in nucleotide or amino acid transport and metabolism; (iii) proteins involved in energy production and conversion; (iv) proteins related to transcription and translation. No Cluster of Orthologous Group (COG) proteins, involved in cell control or cell division, showed differences among the four strains; these proteins are over-represented in *B. longum *NCC2705 [16]. This was not surprising because the bacteria were grown in a rich medium so that stress was minimal. In addition, the proteins in the bifidobacterial shunt pathway, which is a characteristic pathway of the *Bifidobacterium *genus, were well conserved among all strains.

### Differences in cell wall, membrane and envelope biogenesis proteins in the *B. longum *strains

**Figure 2:**
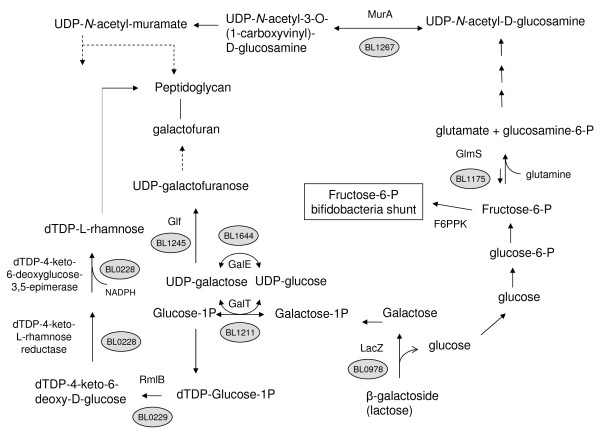
**Schematic representation of peptidoglycan and exopolysaccharide production. Proteins present or absent in the *B. longum *strains are indicated using *B. longum *NCC2705 identification code**.

Of the 38 identified proteins, nine were directly or indirectly linked to cell wall/membrane/envelope biogenesis (Figure 2). Five proteins (BL0228, BL0229, BL1175, BL1245 and BL1267) were directly involved in cell wall/membrane/envelope biogenesis and include the following: dTDP-4-keto-L-rhamnose reductase/dTDP-4-keto-6-deoxyglucose-3,5-epimerase (BL0228), a dTDP-glucose 4,6-dehydratase (RmlB1) (BL0229), a glutamine fructose-6-phosphate transaminase (GlmS) (BL1175), a UDP-galactopyranose mutase (Glf) (BL1245) and a carboxyvinyltransferase (MurA) (BL1267). In addition, two of the identified proteins were involved in carbohydrate metabolism, which is important for cell wall biogenesis: a β-galactosidase (LacZ) (BL0978) and a galactose-1-phosphate uridyltransferase (GalT) (BL1211). Finally, two spots corresponded to proteins indirectly linked to cell wall structure: cyclopropane fatty acid (CFA) synthase (BL1672) and bile salt hydrolase (BSH) (BL0796).

Two of these proteins, BL0229 and BL0228, were detected only in the NCC2705 proteome pattern (Additional file 1 and 2). These proteins play a role in peptidoglycan biogenesis by producing rhamnose, a polysaccharide component of the *Bifidobacterium *peptidoglycan [31]. Rhamnose is synthesized by a *de novo *biosynthetic pathway that starts with dTDP-glucose and leads to the formation of dTDP-L-rhamnose via dehydration and epimerase/reductase reactions mediated by RmlB1 dTDP-glucose 4,6-dehydratase and BL0228 dTDP-4-keto-6-deoxyglucose-3,5-epimerase/dTDP-4-keto-L-rhamnose reductase, respectively [31] (Figure 2). These two enzymes are encoded by genes belonging to the same operon, which is located just downstream of a gene coding for a hypothetical transmembrane protein that may be involved in polysaccharide biosynthesis (BL0230). Interestingly, glutamine fructose-6-phosphate transaminase GlmS (BL1175) was detected in NCC2705 as well as in BS49. GlmS links the D-fructose-6-phosphate shunt of bifidobacteria to the early steps of the *de novo *amino acid sugar biosynthetic pathway, a pathway that is important for the synthesis of cell wall peptidoglycan precursors.

The proteins MurA (BL1267) and Glf (BL1245) were not detected in the BS64 cytosolic proteome. Both proteins are involved in peptidoglycan biosynthesis. MurA is directly linked to the transformation of *N*-acetylglucosamine in that MurA catalyses the first committed step of its incorporation into the peptidoglycan (Figure 2). Meanwhile, Glf catalyzes the ring contraction of UDP-galactopyranose to UDP-galactofuranose, which is then used to form the galactofuran structures that are incorporated into the peptidoglycan (Figure 2).

The spot corresponding to β-galactosidase (*lacZ*, BL0978) was present in *B. longum *NCC2705 and BS89, but not in strains *B. longum *BS49 and BS64. When grown on LB agar medium supplemented with X-gal, β-galactosidase activity was observed not only in NCC2705 and BS89, but also in the BS49 strain (data not shown). This suggests that β-galactosidase activity might be repressed in BS64 and that BS49 may use an enzyme other than BL0978 to metabolize X-gal. The latter is consistent with the observation that several β-galactosidase-encoding genes are predicted in the *B. longum *NCC2705 genome (BL1168 and BL0259). It is noteworthy that the β-galactosidase LacZ is a saccharolytic enzyme, explaining the adaptation of *Bifidobacterium *to its ecological niche, e.g., digestion of complex carbohydrates that escape digestion in the human gastrointestinal tract. In fact, *Bifidobacterium *β-galactosidases show transgalactosylation activity resulting in the production of galacto-oligosaccharides, which are considered prebiotics [32]. The protein differences observed between the four strains may thus reflect different sugar utilization mechanisms that might confer different beneficial properties for the host in terms of probiotic and/or prebiotic activity.

The Leloir pathway enzyme GalT (BL1211) was observed in BS89 and BS49. This enzyme is involved in the UDP-glucose and galactose metabolism that links the anabolic pathway of carbohydrate synthesis to cell wall components and to exopolysaccharide synthesis; galactosides are frequently used as building blocks for exopolysaccharides. Indeed, UDP-galactose is one biosynthetic donor of the galactopyranosyl unit to the galactoconjugates that make up the surface constituents of bacteria, e.g., peptidoglycan (Figure 2) [33,34].

Cyclopropane fatty acid (CFA) synthase (BL1672) was detected only in the NCC2705 strain. Interestingly, CFA synthase is directly linked to modifications in the bacterial membrane fatty acid composition that reduce membrane fluidity and helps cells adapt to their environment [35].

### Proteins with changes in mobility

Mass spectrometry analysis revealed that 12 spots, representing 6 proteins, showed changes in mobility due to charge changes (Additional file 1 and 2). These proteins included a hypothetical protein of unknown function (BL1050), a probable UDP-galactopyranose mutase (Glf) (BL1245), elongation factor Ts (BL1504), a transcription elongation factor (NusA) (BL1615), an UDP-galactopyranose mutase (GalE) (BL1644) and the adenylosuccinate lyase (PurB, BL1800). All had pIs that clearly differed from corresponding proteins in *B. longum *NCC2705. In addition, four spots were identified as different isoforms of the BSH. However, the post-transcriptional modifications leading to the mobility differences are unknown.

### Biological variability among *B. longum *strains

**Figure 3:**
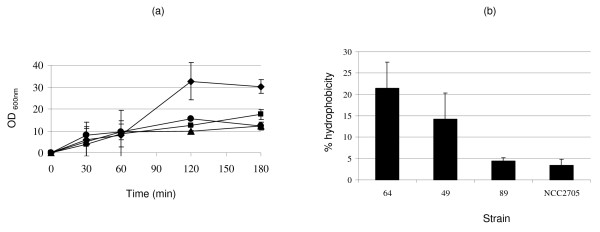
**Aggregation (a) and cell surface hydrophobicity (b) of *B. longum *NCC2705 (black circle), BS64 (black diamond), BS89 (black triangle) and BS49 (black square)**.

Among the 29 spots that differed (present/absent) between the NCC2705 and BS64 proteomes, only 11 proteins from BS64 had an orthologous gene in NCC2705. Comparison of the BS49 and BS89 proteomes to the NCC2705 proteome showed 23 and 26 differences, of which 22 and 14 proteins, respectively, could be identified by comparison to the NCC2705 genome database. Moreover, in BS64, missing spots were identified as enzymes directly or indirectly involved in cell wall/membrane/envelope biogenesis, as noted above. This suggested that BS64 and NCC2705 might show some biological differences in terms of the cell wall properties. To investigate this hypothesis, we compared the surface hydrophobicity of the four strains and their ability to aggregate; these traits reflect the cell surface properties of the strains [36]. Interestingly, BS64 showed three times more autoaggregation than NCC2705 (Figure 3a) and the surface hydrophobicity of BS64 was three times higher than that of NCC2705 (Figure 3b). Because autoaggregation and surface hydrophobicity may impact intestinal colonization, these observations suggest that BS64 and NCC2705 may have different adhesion capabilities. It also suggests possible differences in peptidoglycan between the strains, since peptidolycan is the principal constituent of the bacterial outer membrane that directly contacts the surrounding environment. Adhesion of bifidobacteria to the gastrointestinal epithelium plays an important role in colonization of the gastrointestinal tract and provides a competitive advantage in the ecosystem against pathogens.

## Conclusion

This study used proteomics to analyze cytosolic proteins extracted from four strains of bifidobacteria grown in a rich laboratory medium. The results validated proteomics as a tool for exploring the natural diversity and biological effects of bifidobacteria. Specifically, proteomics allowed identification of phenotype differences in *B. longum *strains that have different in vitro properties. Interestingly, by comparing 2D-electrophoresis patterns and by identifying proteins that were present in some strains but not others, we found that the protein diversity observed between the strains was related to differences in cell wall/membrane biogenesis. In one of the strains (BS64), it was associated with better autoaggregation and greater surface hydrophobicity. This strain has been reported to be an inducer of T-helper 2 cytokines; in contrast, NCC2705 had the lowest surface hydrophobicity of the four strains and has been reported to induce T-helper 1 cytokines [28]. This study showed that proteomic approach may help researchers understand the differential effects of bifidobacteria and be useful for identifying bifidobacteria with probiotic potential.

## Methods

### Strains, media and growth conditions

*B. longum *NCC2705 was kindly provided by the Nestlé Research Center (Lausanne, Switzerland). *B. longum *CUETM 89-215 (BS89), BS49 and BS64 were isolated from the dominant fecal flora of healthy infants [28]. Strains were cultured on Wilkins-Chalgren anaerobe agar (Oxoid) supplemented with 1% (w/v) D-glucose, 0.05% (w/v) L-cysteine, 0.5% (v/v) Tween 80 (WCB) and incubated for 48 hrs at 37°C in a chamber under anaerobic conditions (CO_2_:H_2_:N_2_, 10:10:80). After genomic DNA extraction, *Bifidobacterium *strains were identified by multiplex PCR and amplification and sequencing of the 16S rRNA, as previously described [37].

TGYH broth (tryptone peptone, 30 g l^-1^; glucose, 5 g l^-1^; yeast extract, 20 g l^-1^; haemin, 5 g l^-1^) was used for cell growth prior to protein extraction. Three independent growth experiments were performed for each strain to extract cytosolic proteins. β-galactosidase activity was visualized on Luria-Bertani (LB) (Oxoid) agar plates supplemented with X-gal (40 mg l^-1^).

### Genotyping using PFGE

PFGE was performed as previously described using the XbaI restriction enzyme [29]. Gels were run using a clamped homogeneous electric-field apparatus (CHEF-DRIII, Bio-Rad), and *Staphylococcus aureus *NCTC 8325 DNA was used as a reference. GelCompar software (Bio-Rad) was used for cluster analysis (Applied Maths) with the Dice correlation coefficient, and a dendrogram was produced with the unweighted pair-group method using the arithmetic averages clustering algorithm.

### Cytosolic protein extraction and 2D-electrophoresis

Cytosolic cell extracts were obtained from 300 ml of culture in TGYH medium that was collected at the mid-log exponential growth phase (OD_600 _of 0.8-0.9). Cytosolic protein extraction and 2D-electrophoresis were performed as previously described [21]. The protein concentration of each bacterial extract was measured using the Coomassie Protein Assay Reagent kit (Pierce Biotechnology) according to the manufacturer's instructions. For electrophoresis, proteins from bifidobacterial extracts (350 μg) were loaded onto strips (17 cm) with a pH range of 4 to 7 (Bio-Rad), focused for 60,000 V·h, and the second dimension was carried out using a 12.5% SDS-polyacrylamide gel. The gels were stained with Bio-Safe Coomassie (Bio-Rad). Spot (present in all replicates) detection was carried out using Progenesis SameSpots software (Nonlinear Dynamics) and a master gel image was produced. The reproducibility of spot differences was confirmed by analyzing three gels for each strain, each obtained using an independent culture.

Spots of interest were subjected to tryptic in-gel digestion and identified by matrix-assisted laser desorption ionization-time of flight mass spectrometry (MALDI-TOF/MS) using a Voyager DE STR Instrument (Applied Biosystems), as previously described [38]. The α-cyano-4-hydroxycinnamic acid matrix was prepared at 4 g l^-1 ^in 0.1% TFA, 50% acetonitrile. An equal volume (1 μl) of matrix and sample were spotted onto the MALDI-TOF target plate. Spectra were acquired in the reflector mode with the following parameters: 2250 laser intensity, 20 kV accelerating voltage, 62% grid voltage, 135 ns delay. The mass gates used were 700-4000 Da. Internal calibration was performed by using the trypsin peptides at 842.5 and 2211.1 Da. Spots mass accuracy varied between 15-30 ppm. The carbamidomethylation of cysteines, methionine oxidation and one miscleavage were considered during the search. A minimum of four matching peptides and a sequence coverage above 25% were required before considering this a result of the database search. Additional parameters were used to assume a correct identification: theoretical molecular weight and isoelectric point in good agreement with experimental values.

Proteins were identified using MS-Fit software (University of California San Francisco Mass Spectrometry Facility; http://prospector.ucsf.edu and Mascot software (Matrix Science Inc., Boston, MA; http://www.matrixscience.com). The genome database entries of the chromosome of *B. longum *NCC2705 (GenBank database accession no. AE014295) were used to assign putative genes encoding the cytosolic proteins of interest from the four *B. longum *extracts using peptide mass fingerprinting. Based on comparison against the master gel, we identified spots that were not present in all strains, i.e. pattern differences. The presence or absence of a spot (protein) can reflect whether the gene encoding the protein is present, is expressed or repressed, or may reflect a change in the location of the spot on the gel. Our approach resulted in identification of spots (proteins) corresponding to genes in the NCC2705 genome.

### Aggregation and cell surface hydrophobicity assays

The aggregation assay was performed using bacteria grown at 37°C for 48 hrs in TGYH broth that was harvested and resuspended in TGYH at an OD_600 _of 0.5. During incubation at 37°C, the OD_600 _of the suspension was monitored at 30, 60, 120 and 180 min, and aggregation was expressed as [1-(OD_600 _upper suspension/OD_600 _total bacterial suspension)] × 100 [36]. To assay cell surface hydrophobicity, bacteria were grown in TGYH as described above, washed twice in 10 ml phosphate buffer (pH 6.5, 50 mM) and diluted in the same buffer to OD_600 _= 1. This bacterial suspension (2 ml) was added to an equal volume of xylene and mixed for 2 min by vortexing. The OD_600 _was measured. Cell surface hydrophobicity (H) was calculated as follows: [(1-OD_aqueous phase_)/OD_initial_] × 100 [39].

## Authors' contributions

JA performed the PFGE, proteomic and phenotype experiments. PA helped design the study and performed protein spot detection using Progenesis SameSpot software. FB prepared samples for MALDI-TOF/MS and identified proteins using protein identification software. JA, MZ, MCCV and MJB conceived the study, participated in the study design process, and helped write the manuscript. All authors read and approved the final manuscript.

## Supplementary Material

Additional file 1 **Table S1- Identification of selected protein spots that showed variation (presence/absence) among the *B. longum *NCC2705, BS49, BS89 and BS64 strains**. Additional file 1 contains Table S1 where are presented spot identification and characteristics. Click here for file

Additional file 2 **2D-electrophoretic gel of *B. longum *NCC2705, BS49, BS89 and BS64 cytosolic proteins. Spots that are present in some strains and absent in others are highlighted. Spot characteristics are listed in Table S1**. Additional file 2 contains 2D-electrophoretic gel pictures of *B. longum *NCC2705, BS49, BS89 and BS64 cytosolic proteins. Click here for file
