# Supplementary figures and images for: Proteomic comparison of the cytosolic proteins of three Bifidobacterium longum human isolates and B. longum NCC2705
Source: BMC Microbiol. 2010 Jan 29;10:29. doi: 10.1186/1471-2180-10-29 (PMC2824696; doi:10.1186/1471-2180-10-29)

## Slide 1
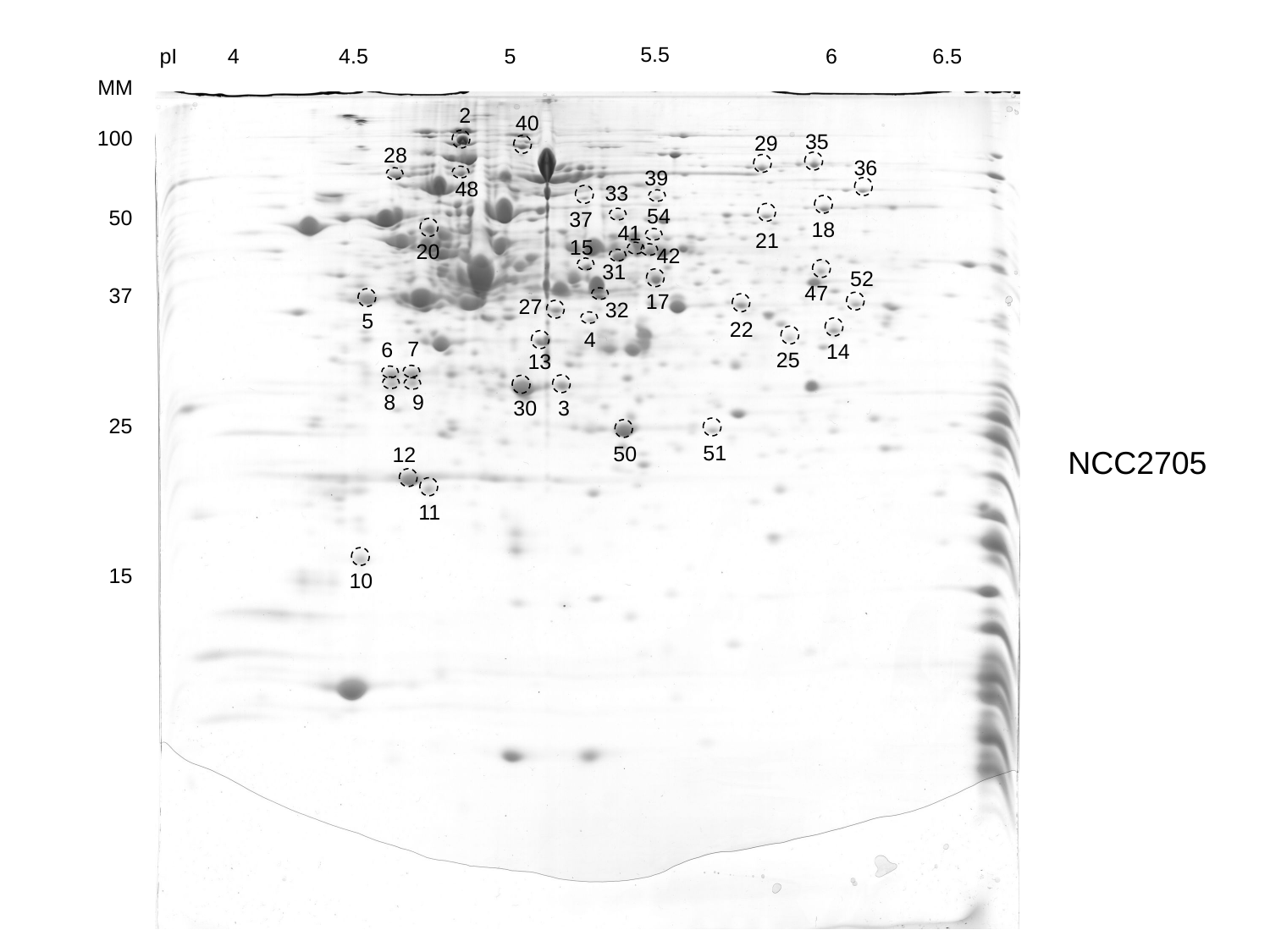

5.5
4
4.5
5
6
6.5
pI
MM
2
40
100
35
29
28
36
39
48
33
54
50
37
18
41
21
15
20
42
31
52
47
37
17
27
32
5
22
4
7
6
14
25
13
8
9
3
30
25
51
50
12
NCC2705
11
15
10

## Slide 2
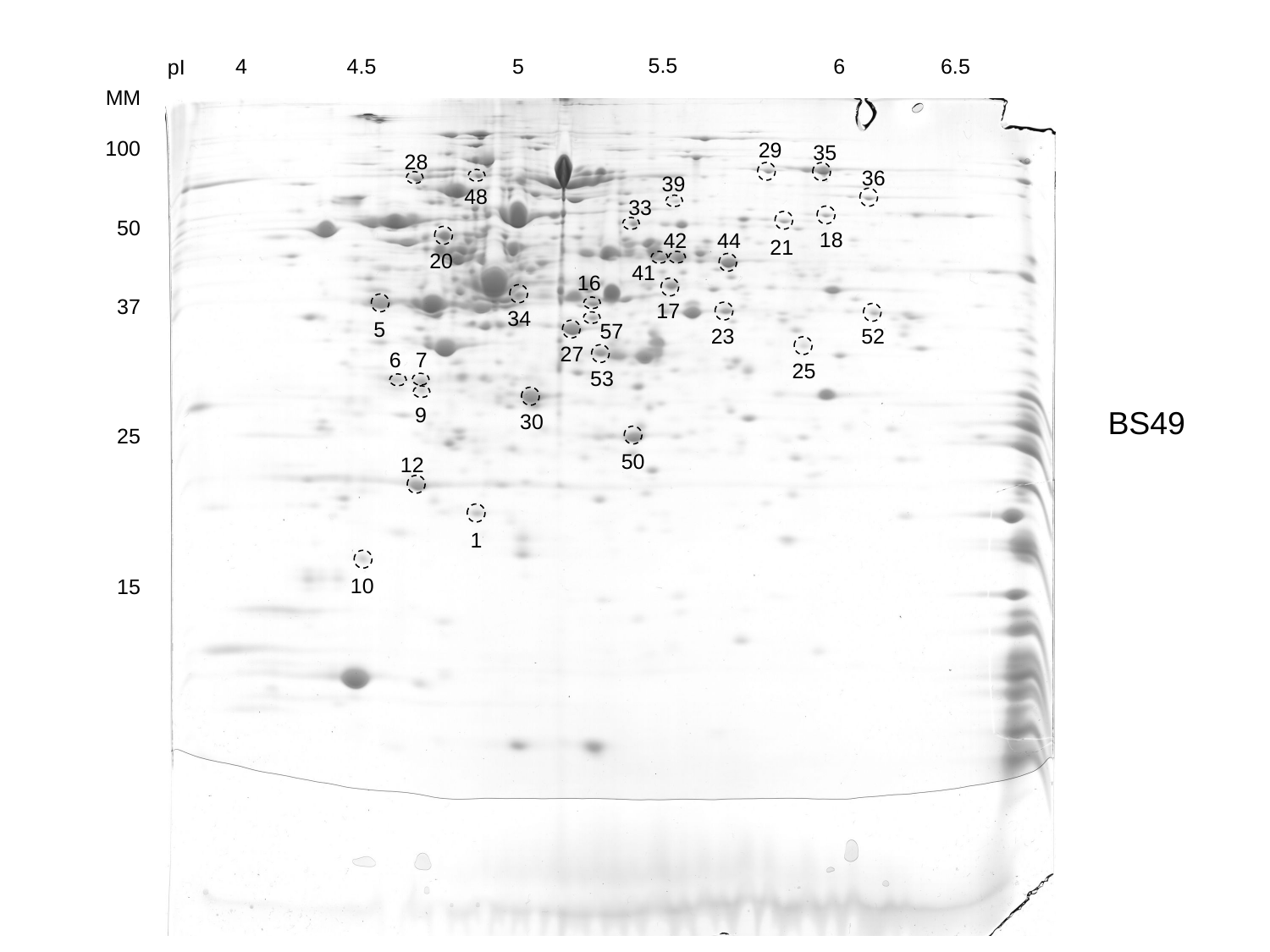

5.5
4
4.5
5
6
6.5
pI
MM
100
29
35
28
36
39
48
33
50
18
42
44
21
20
41
16
37
17
34
5
57
23
52
27
7
6
25
53
9
BS49
30
25
50
12
1
10
15

## Slide 3
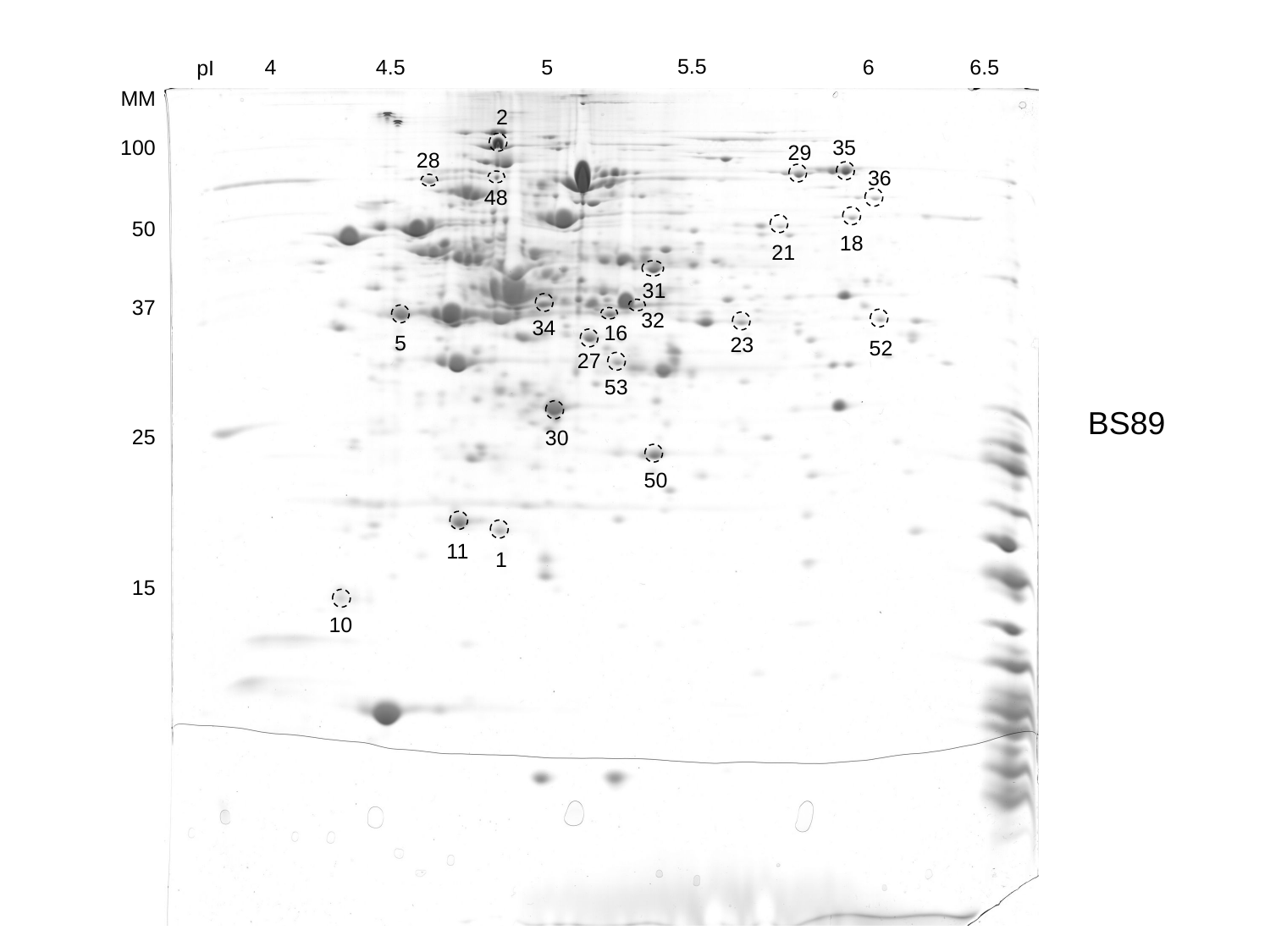

5.5
4
4.5
5
6
6.5
pI
MM
2
35
100
29
28
36
48
50
18
21
31
37
32
34
16
5
23
52
27
53
BS89
25
30
50
11
1
15
10

## Slide 4
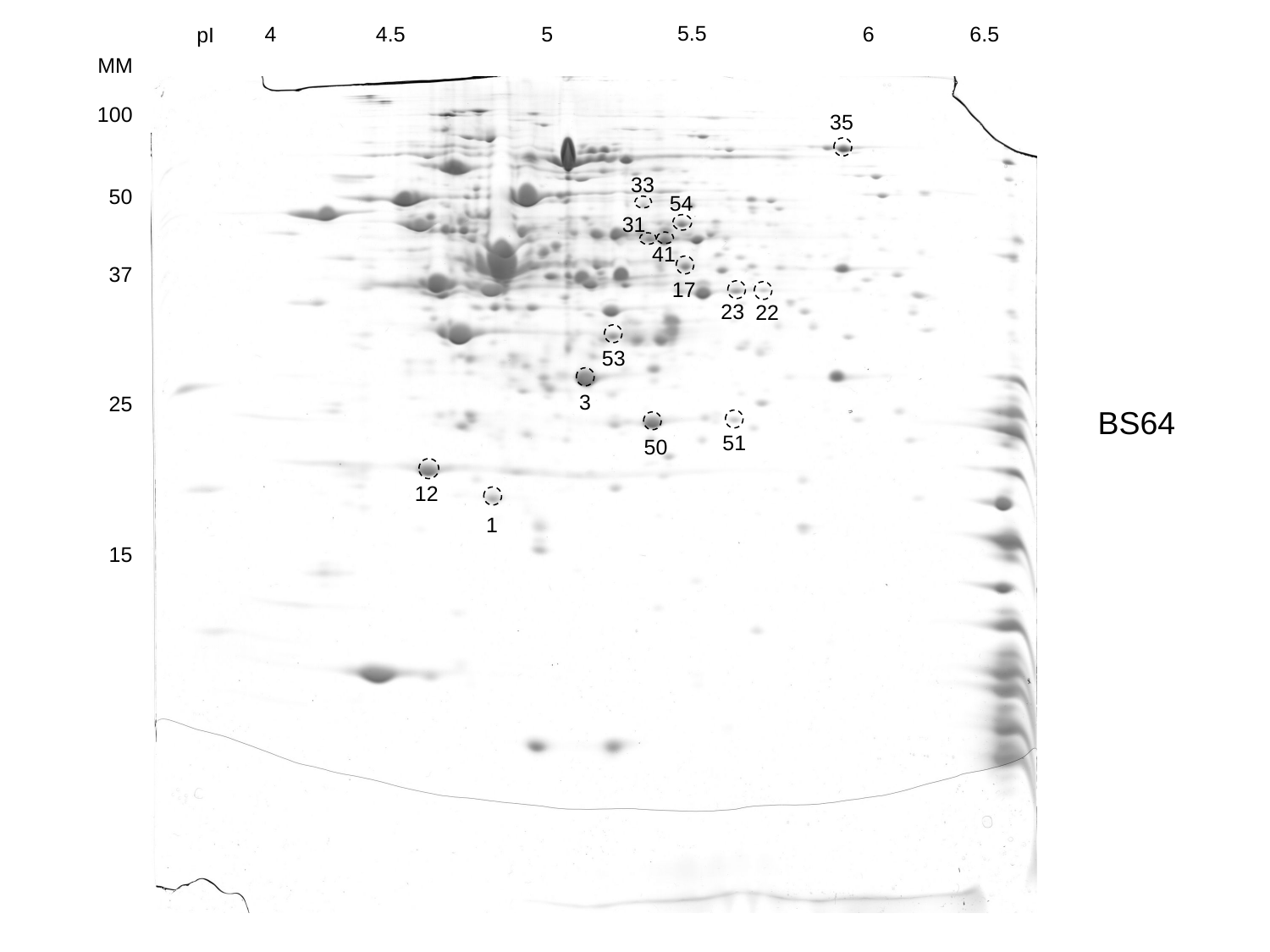

5.5
4
4.5
5
6
6.5
pI
MM
100
35
33
50
54
31
41
37
17
23
22
53
3
25
BS64
51
50
12
1
15

Supplement: Additional file 2 — 2D-electrophoretic gel of B. longum NCC2705, BS49, BS89 and BS64 cytosolic proteins. Spots that are present in some strains and absent in others are highlighted. Spot characteristics are listed in Table S1. Additional file 2 contains 2D-electrophoretic gel pictures of B. longum NCC2705, BS49, BS89 and BS64 cytosolic proteins. [file 1471-2180-10-29-S2.PPT]
